# Correlation Between Gut Microbiota Shifts, Lipopolysaccharide Levels, and Neuroinflammatory Responses Following Ischemic Stroke

**DOI:** 10.3390/cells15191819

**Published:** 2026-10-06

**Authors:** Kemin Li, Wancong Ding, Shuang Wang, Gaowei Pan, Shengxiang Zhang

**Affiliations:** 1 School of Food Science and Engineering, Xinjiang Institute of Technology, No. 1 Xuefu Road West, Aksu 843100, China; 2 Gansu Key Laboratory of Biomonitoring and Bioremediation for Environmental Pollution, School of Life Sciences, Lanzhou University, No. 222 South Tianshui Road, Lanzhou 730000, China; dingwc2023@lzu.edu.cn (W.D.); wshuang2025@lzu.edu.cn (S.W.); pangw20@lzu.edu.cn (G.P.)

**Keywords:** microbiota–gut–brain axis (MGBA), ischemic stroke, lipopolysaccharide (LPS), neuroinflammatory responses, microglial polarization

## Abstract

**Highlights:**

**What are the main findings?**

- Ischemic stroke is accompanied by elevated LPS levels in the gut, circulation, and brain.
- Changes in gut *Verrucomicrobiota* abundance are spatially associated with increased LPS immunoreactivity in the ischemic brain.

**What are the implications of the main findings?**

- These findings reveal a correlation between gut microbial shifts, circulating LPS, and cerebral LPS immunoreactivity, providing an observational foundation for future mechanistic studies.
- LPS may serve as a correlative marker linking gut microbiota changes to neuroinflammation after ischemic stroke.

**Abstract:**

Background: The microbiota–gut–brain axis (MGBA) is increasingly recognized as a contributor to ischemic stroke pathophysiology, yet the relationship between gut microbial changes and neuroinflammation remains incompletely understood. This study aimed to investigate whether shifts in gut microbiota are associated with concurrent changes in lipopolysaccharide (LPS) levels and neuroinflammatory responses following ischemic stroke. Methods: Using a photothrombotic mouse model, we measured LPS in the gut, serum, and brain via ELISA and immunohistochemistry. Gentamycin was administered to deplete gut bacteria and assess its effects on LPS and stroke recovery. Upon TLR4 inhibition with TAK-242, microglial polarization and inflammatory cytokines were evaluated, and dose-dependent LPS supplementation was performed. Results: Ischemic stroke was accompanied by elevated LPS levels in the gut, circulation, and brain, paralleling *Verrucomicrobiota* expansion. Antibiotic treatment reduced *Verrucomicrobiota* and LPS in the gut and brain, and this was associated with worsened stroke outcomes. TLR4 inhibition was associated with altered microglial phenotypes and inflammatory markers. Exogenous LPS produced dose-dependent effects, whereas endogenous LPS levels remained low. Conclusions: These findings reveal a spatial correlation between gut microbial shifts, circulating LPS, and cerebral LPS immunoreactivity, suggesting LPS may serve as a correlative marker of gut–brain interactions following ischemic stroke.

## 1. Introduction

Up to now, ischemic stroke remains the major cause of mortality and disability worldwide [1]. Ischemic insult can lead to injuries of the central nervous system (CNS), including neuronal damage, blood–brain barrier (BBB) disruption, as well as tissue necrosis, and the disorders are mostly irreversible [2,3,4]. Apart from the direct damage to the CNS, stroke can even disrupt gut homeostasis [5]. For instance, ischemic stroke reduces the expression of intestinal tight-junction proteins and further leads to leakage from the intestines [6]. The composition of gut microbiota is significantly altered after stroke [7]. In turn, microbiota dysbiosis also affects stroke outcomes [8,9,10]. For example, depletion of gut microbiota by antibiotic pretreatment worsens stroke outcomes [11]. Transplantation of fecal microbiota from healthy donors has been shown to exert neuroprotective effects following stroke [12,13]. However, the roles and mechanisms of gut microbiota in ischemic stroke remain largely unknown.

Recent evidence has suggested that gut microbial metabolites play essential roles in the onset and progression of various CNS disorders, including ischemic stroke [14,15,16,17]. For example, trimethylamine-N-oxide (TMAO), as a waste product of microbiota in the gut, its accumulation can cause atherosclerosis and can be used as a biomarker for the risk of stroke occurrence and recurrence [18,19]. In addition, short-chain fatty acids (SCFAs) produced by intestinal bacteria are significantly reduced after stroke, and SCFA supplementation improved the outcomes of stroke [20,21]. Apart from these metabolites, the components of gut microbiota also play major roles in the MGBA [22]. For example, LPS plays key roles in host–pathogen interactions with the innate immune system [23]. Previous studies have shown that LPS levels in the plasma are significantly increased in both Alzheimer’s disease (AD) patients and animal models, and LPS is demonstrated to be abundant in AD-affected brain neocortex and hippocampus [24]. However, the roles and underlying mechanisms of microbial compositions in neurodegenerative diseases remain to be elucidated.

Here, we investigated the association between gut microbiota, LPS, and neuroinflammatory responses following ischemic stroke. Following photothrombosis-induced ischemic stroke, LPS levels were significantly elevated in the gut, blood, and brain. Gentamycin treatment significantly reduced the abundance of *Verrucomicrobiota* and the levels of LPS in the gut and brain, along with worsened stroke outcomes, whereas serum LPS showed no significant change. Inhibition of the LPS/TLR4 pathway using TAK-242 shifted microglial polarization toward a pro-inflammatory phenotype and impaired recovery after ischemic stroke. LPS supplementation confirmed that its effects were dose-dependent, whereas endogenous LPS levels remained relatively low under ischemic conditions. Collectively, our findings reveal a significant association between gut microbial changes, systemic LPS elevation, and post-stroke neuroinflammation, highlighting LPS as a potential link within the MGBA.

## 2. Materials and Methods

### 2.1. Mice

CX3CR1^GFP/+^ mice, in which microglia are labeled with green fluorescent protein (GFP), were used to identify microglia in immunofluorescence experiments [25]. Male C57BL/6 mice aged 8–10 weeks (18–24 g) served as wild-type controls. GFP expression was used to identify microglia in brain tissue. Mice were sourced from the Jackson Laboratory and reared in the Core Facility of the School of Life Sciences, Lanzhou University. The animal study was reviewed and approved by the Ethics Committee of Lanzhou University, China (Approval No. EAF2022037).

### 2.2. Photothrombosis Stroke Model

The rose bengal photothrombosis ischemic stroke model was applied in this work [26,27]. In brief, mice received intraperitoneal injections of ketamine (150 mg/kg) and xylazine (25 mg/kg) for anesthesia. The location of the cranial window (2 mm diameter) is over the right somatosensory cortex and at coordinates of −1.5 mm from bregma and 1.5 mm lateral. The thickness of the skull was reduced to 50 μm by a high-speed cranial drill. The rose bengal (0.03 mg/g mouse; Sigma, St. Louis, MO, USA) was injected intravenously into the tail vein. Green light (535 ± 25 nm; mercury lamp with a 535 nm bandpass filter) was applied to the cortical surface for 5 min over a 1 mm^2^ region to achieve photoactivation. Subsequent experiments were performed only on mice showing evident thrombus formation and reduced blood flow within 5 min.

### 2.3. Two-Photon Imaging

To image the blood vessels pre- and post-photothrombosis, two-photon imaging was applied in this study. Briefly, mice were deeply anesthetized, and the skull was thinned to ~25 μm by using a high-speed dental drill. Rose bengal (0.03 mg/g mouse; Sigma) was administered via tail vein injection to label the vasculature. The cranial window of rose bengal photothrombosis was exposed to a water immersion objective lens (25×/1.05; Olympus, Tokyo, Japan). The laser was adjusted to 920 nm, and the Z step size was acquired as repeated Z-series of 30 to 50 optical sections (1024 × 1024 pixel arrays) with a step size of 1 μm. The images were analyzed by using ImageJ software (version 1.53).

### 2.4. Gut Microbiome Analysis

After brief restraint, fecal pellets were obtained from each mouse. The tail was raised, and gentle pressure was applied to the lower abdomen to induce defecation. Feces were then transferred with sterile forceps into sterile tubes and preserved at −80 °C. Each specimen contained 2–3 pellets. Gut microbiome changes after ischemic stroke were evaluated by 16S rDNA amplicon sequencing (LC-Bio Technologies, Hangzhou, China). The OMEGA Stool DNA Kit (OMEGA Bio-tek, Inc., Norcross, GA, USA) was used for microbial DNA extraction. Primers targeting conserved regions were designed to amplify the variable region of the 16S/ITS2 rDNA gene. Following detection via 1.5% agarose gel electrophoresis, target fragments were recovered with the AxyPrep PCR Cleanup Kit (Axygen Biosciences, Union City, CA, USA), and further purification was carried out using the Quant-iT PicoGreen dsDNA Assay Kit (ThermoFisher Scientific, Waltham, MA, USA). Library quantification was performed using the Promega QuantiFluor fluorescence system (Promega Corporation, Madison, WI, USA), and the pooled library was loaded onto an Illumina platform for paired-end sequencing (2 × 250 bp). Paired-end reads were subsequently assigned to individual samples based on barcode sequences, which were then removed along with primer sequences. Reads were merged using FLASH (v1.2.8) or PEAR (v0.9.6). Quality filtering was applied with fqtrim (v0.94) to obtain high-quality clean tags, and chimeric sequences were eliminated using Vsearch (v2.3.4). Following dereplication via DADA2, a feature table and corresponding sequences were generated. Alpha and beta diversity metrics were calculated using QIIME2 (version 2020.11), and bacterial taxonomic relative abundances were analyzed. Species annotation was conducted through sequence alignment using Blast against the SILVA and NT-16S databases. Visualizations were created with R software packages (v3.5.2). The sequencing data are accessible under accession numbers PRJNA990935 and PRJNA1073280.

### 2.5. Pharmacological Administration

#### 2.5.1. Administration of TAK-242

The TLR4 antagonist TAK-242 (M4838; AbMole BioScience, Inc., Houston, TX, USA) was used to inhibit LPS/TLR4 signaling pathway [28]. TAK-242 (0.12 mg/kg mouse; 0.8 mg/mL in saline containing 1% DMSO) was injected into the left ventricle prior to the establishment of stroke modeling. The control group received an equivalent volume of 1% DMSO in saline via the same route. Briefly, mice were fixed on the stereotaxic apparatus (DW-2000; Chengdu Techman Software Co., Ltd, Chengdu, China) after deep anesthesia. The location of injection was at coordinates of −0.46 mm from bregma, −1.0 mm lateral and the depth of needling was 2.7 mm. The drug solution was infused at a rate of 0.5 μL/min using a microsyringe. The needle was left in place for 5 min before infusion and remained for an additional 10 min after completion of the injection to allow for drug diffusion and to prevent backflow, after which the needle was slowly withdrawn.

#### 2.5.2. LPS Treatment

Based on the detected concentration of LPS (about 5 ng/mL, and the volume of blood in an adult mouse was approximately 2 mL) in serum after ischemic stroke, mice were injected intravenously with LPS (L8880; Beijing Solarbio Science & Technology Co., Ltd, Beijing, China) at the concentration of 10 ng/mouse, 50 ng/mouse, and 100 ng/mouse before the establishment of the stroke model.

#### 2.5.3. Administration of Gentamycin Sulfate

As an antibiotic that is effective against Gram-negative bacteria, gentamycin sulfate (G6064; Shanghai Macklin Biochemical Co., Ltd, Shanghai, China) was used to inhibit Gram-negative bacteria in the gut [29]. Mice were treated with gentamycin sulfate (0.4 mg/g mouse; diluted to 40 mg/mL in normal saline) by oral gavage twice daily for 4 days before the stroke modeling.

### 2.6. Immunofluorescence (IF) Staining

Mice were anesthetized with an overdose of urethane (2.25 mg/g mouse), followed by transcardial perfusion with 0.1 M PBS and 4% paraformaldehyde (PFA) in PBS. The brains were then removed and soaked in 4% PFA for 48 h. Next, they were sectioned into 30 μm coronal sections using a vibratome (VT 1000 S, Leica Biosystems, Wetzlar, Germany). The following primary antibodies were used in this study: anti-*Escherichia coli* LPS (1:100; Abcam plc, Cambridge, UK, ab35654), TLR4 polyclonal antibody (1:300; Proteintech, Wuhan Sanying, Wuhan, China), iNOS polyclonal antibody (1:300; Proteintech), and anti-Arg1 antibody (1:300; Boster Biological Technology Co., Ltd, Wuhan, China). IF staining was performed by initially incubating brain sections with primary antibodies overnight at 4 °C. Sections were then incubated for 2 h at room temperature with secondary antibodies, including rhodamine-conjugated Affinipure goat anti-mouse and goat anti-rabbit (both from Proteintech, 1:200). After washing, sections were mounted using a medium composed of 70% 0.1 M PBS and 30% glycerol. Images were captured on a confocal microscope (FV1000, Olympus Corporation, Tokyo, Japan). All IF images were taken from the infarct border zone (ischemic penumbra) at the cranial window level.

### 2.7. Immunohistochemistry (IHC) Staining

IHC staining was conducted following the procedure previously described in references [30,31,32]. Paraffin sections (6 μm) were mounted on adhesive glass slides and stored at room temperature. The de-waxing was performed using xylene, and the sections were transferred into a gradient of alcohol: 100%, 95%, 80%, and 70%, each for 3 min. Antigen retrieval was achieved by boiling in sodium citrate buffer (pH 6.0) for 20 min. Endogenous peroxidase was blocked using 3% hydrogen peroxide for 30 min in darkness. Blocking buffer containing 10% normal goat serum was applied at 37 °C for 30 min. Subsequently, the sections were incubated overnight at 4 °C with the primary LPS antibody (diluted 1:100). After washing with PBS three times for 5 min each, the sections were incubated with horseradish peroxidase–conjugated affinipure goat anti-mouse antibody (diluted 1:100; proteintech) for 2 h at room temperature, followed by DAB (3,3-N-DiaminobenzidineTertrahydrochloride) staining for 10 min. Finally, sections were counterstained with haematoxylin, and images were captured with an Olympus DP72 microscope (Olympus Corporation, Tokyo, Japan).

### 2.8. Quantitative Real-Time PCR (qRT-PCR) Analysis

Initially, mice were anesthetized with an overdose of urethane (2.25 mg/g mouse) and subsequently underwent transcardial perfusion with 20 mL of cold 0.1 M PBS. RNA was isolated from the infarcted brain regions with RNAiso Plus (0.01 mL/mg; Takara Bio, Inc., Kusatsu, Shiga, Japan). The PrimeScript RT Reagent Kit with gDNA Eraser (Takara) was employed to obtain cDNA. Gene expression was analyzed using qRT-PCR with TB Green^®^ Premix DimerEraser™ (Takara). The primers for the tested genes are listed in Table S1 of the Supplementary Material. Relative mRNA expression was quantified by the 2^−ΔΔ*C_T_*^method, with GAPDH serving as the internal reference.

### 2.9. Enzyme-Linked Immunosorbent Assay (ELISA) of LPS

Blood was harvested and maintained at 4 °C overnight. Following centrifugation (1000× *g*, 20 min), the supernatant was obtained. Serum LPS levels were assessed with the Mouse LPS ELISA Kit (JL20691; Shanghai Jianglai Biological Technology Co., Ltd, Shanghai, China) as described by the manufacturer.

### 2.10. Western Blotting (WB) Analysis

The tissues for the WB were from the ischemic zone. Total protein was extracted by the ultrasonic cell-break method in a lysis buffer (RIPA:pmsf = 100:1). The samples of WB were obtained by boiling the liquid supernatant, which was mixed with loading buffer. The quantification of protein samples was performed by the BCA Protein Quantitative Detection Kit (Servicebio, Wuhan, China). The electrophoresis was performed in a 12% polyacrylamide gel. Then, the separated proteins from the gel were transferred to a PVDF membrane. The PVDF membrane was incubated in a blocking buffer (5% non-fat dry milk in TBS-T) for 1 h at room temperature. Next, the membrane was incubated with the specific primary antibody at 4 °C overnight and then incubated with the secondary antibody for 2 h at room temperature. Antibodies used in this study were Anti-Arginase-1 (1:1000; BM3973, BOSTER), Anti-IL6 (1:1000; BA4339, BOSTER), Anti-TNF-alpha Rabbit pAb (1:600; GB11188, Servicebio), Mouse Anti-β-actin mAb (1:1000; TA-09, ZSGB-BIO, Beijing, China), HRP-conjugated Affinipure Goat Anti-Mouse IgG (H + L) (1:5000; SA00001-1, Proteintech), and HRP-conjugated Affinipure Goat Anti-Rabbit IgG (H + L) (1:5000; SA00001-2, Proteintech). Blots were imaged on a Fusion FX SPECTRA system (VILBER, Paris, France). Band intensities were measured with ImageJ and normalized to β-actin.

### 2.11. Statistical Analysis

Quantification of IHC staining was performed with the IHC Profiler plugin in ImageJ [33]. Three random square fields (300 μm × 300 μm) per stained brain section were chosen for counting iNOS-positive microglia, Arg1-positive microglia, and FJC-positive cells. Analyses were carried out using GraphPad Prism Version 6.02 (La Jolla, San Diego, CA, USA). For the comparisons between two groups, the Mann-Whitney U test was used for all datasets. For the comparisons among three or more groups, one-way ANOVA followed by Tukey’s post hoc test was used. All “*n*” values reported in the figures and figure legends represent the number of biologically independent mice per group. Values are given as mean ± S.E.M. Asterisks denote statistical significance: * *p* < 0.05, ** *p* < 0.01, *** *p* < 0.001, and **** *p* < 0.0001.

## 3. Results

### 3.1. Ischemic Stroke Induces Concurrent Elevation of LPS in the Gut, Blood, and Brain

**Figure 1:**
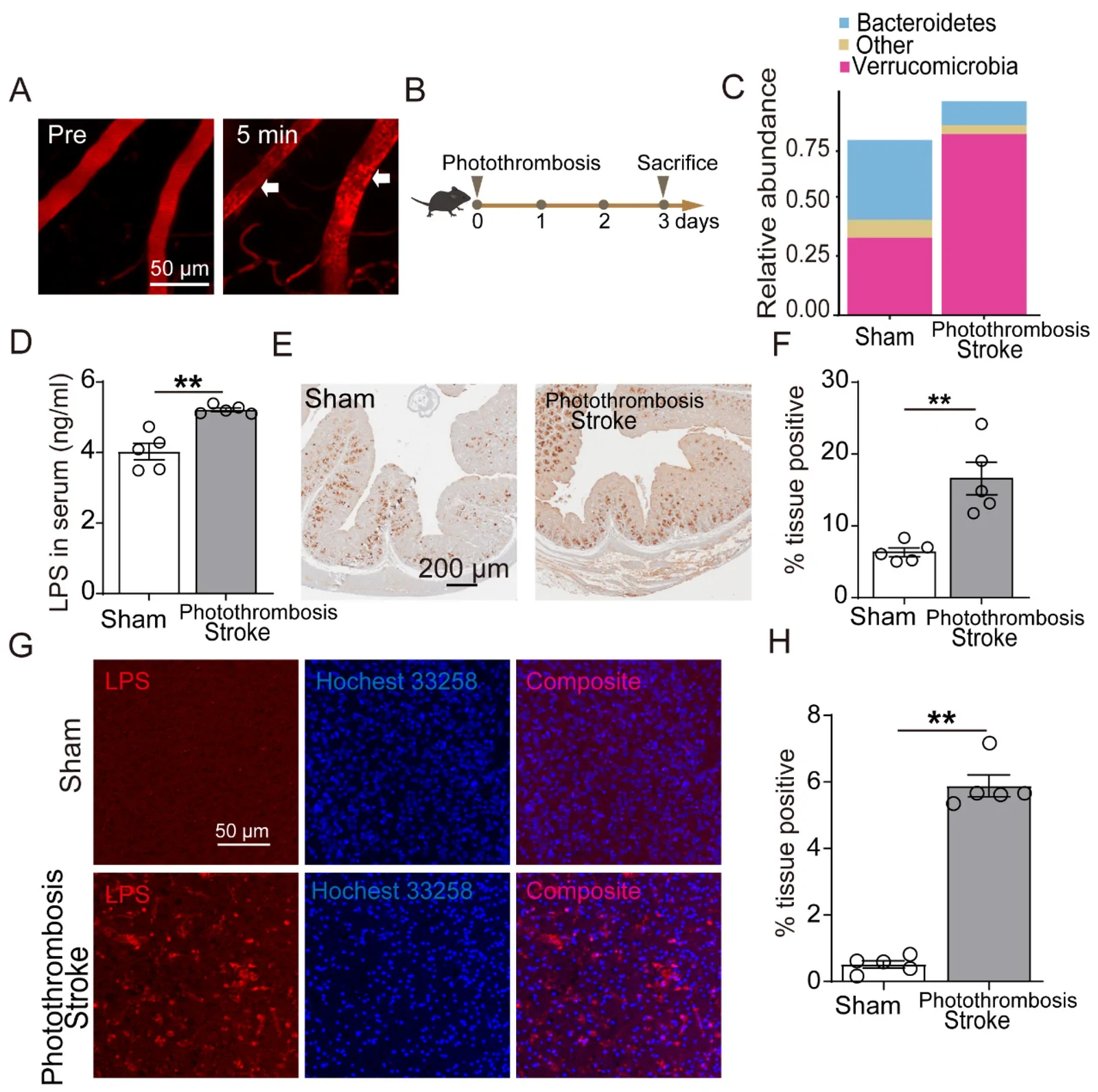
Assessment of LPS levels in the blood, gut, and brain following ischemic stroke. (**A**) Representative two-photon microscopy images of the vasculature after rose bengal administration. Thrombus formation was detected 5 min after green light exposure. Arrows denote vessels obstructed by thrombi. (**B**) Diagram illustrating the experimental workflow. (**C**) BugBase analysis of 16S rDNA amplicon sequencing after ischemic stroke. (**D**) The concentration of LPS in serum in sham and stroke groups was analyzed with the Mann-Whitney U test (*n* = 5 per group). (**E**) IHC staining of LPS on colons. (**F**) Percentage of LPS-positive staining area compared between the sham and stroke groups by the Mann-Whitney U test (*n* = 5 per group) (**G**) Images of brain sections from the infarct border zone immunostained for LPS in sham and stroke groups. (**H**) Quantitative LPS levels in the brain were analyzed with the Mann-Whitney U test (*n* = 5 per group). Data are shown as mean ± S.E.M. ** *p* < 0.01.

To explore gut–brain communication after ischemic stroke, photothrombosis was induced via the rose bengal method, in which the mouse cortex was illuminated with green light for 5 min (Figure 1A). Mice were sacrificed 3 days after photothrombosis (Figure 1B). Firstly, BugBase prediction analysis of 16S rDNA amplicon sequencing revealed a notable increase in *Verrucomicrobiota*, a phylum of Gram-negative bacteria, following ischemic stroke (Figure 1C). Consistent with this finding, we also detected a significant increase in LPS, a major component of Gram-negative bacteria, after ischemic stroke. Next, the concentration of LPS in serum was examined by ELISA, and the data indicated a marked increase in LPS levels in blood after ischemic stroke (4.026 ± 0.215 ng/ml for sham group versus 5.216 ± 0.049 ng/ml for stroke group, Figure 1D). Subsequently, the IHC staining in intestinal tissues was applied to detect LPS level in gut, and the results showed that LPS level was significantly increased after ischemic stroke compared with sham group (6.65% ± 1.65% for sham group versus 18.3% ± 6.6% for stroke group, Figure 1E,F). We further examined LPS immunoreactivity in the brain by IF staining on brain slices. The results showed that there were no visible LPS signals in brain tissues of the sham group, but LPS-positive signals were significantly enhanced in the infarct area of the stroke brain (Figure 1G,H and Figure S1A,B). Together, these data suggest that the increase in gut *Verrucomicrobiota* is accompanied by elevated LPS levels in both the peripheral circulation and the brain following ischemic stroke.

### 3.2. Gentamycin Treatment Reduces LPS Levels in the Gut, Blood, and Brain

**Figure 2:**
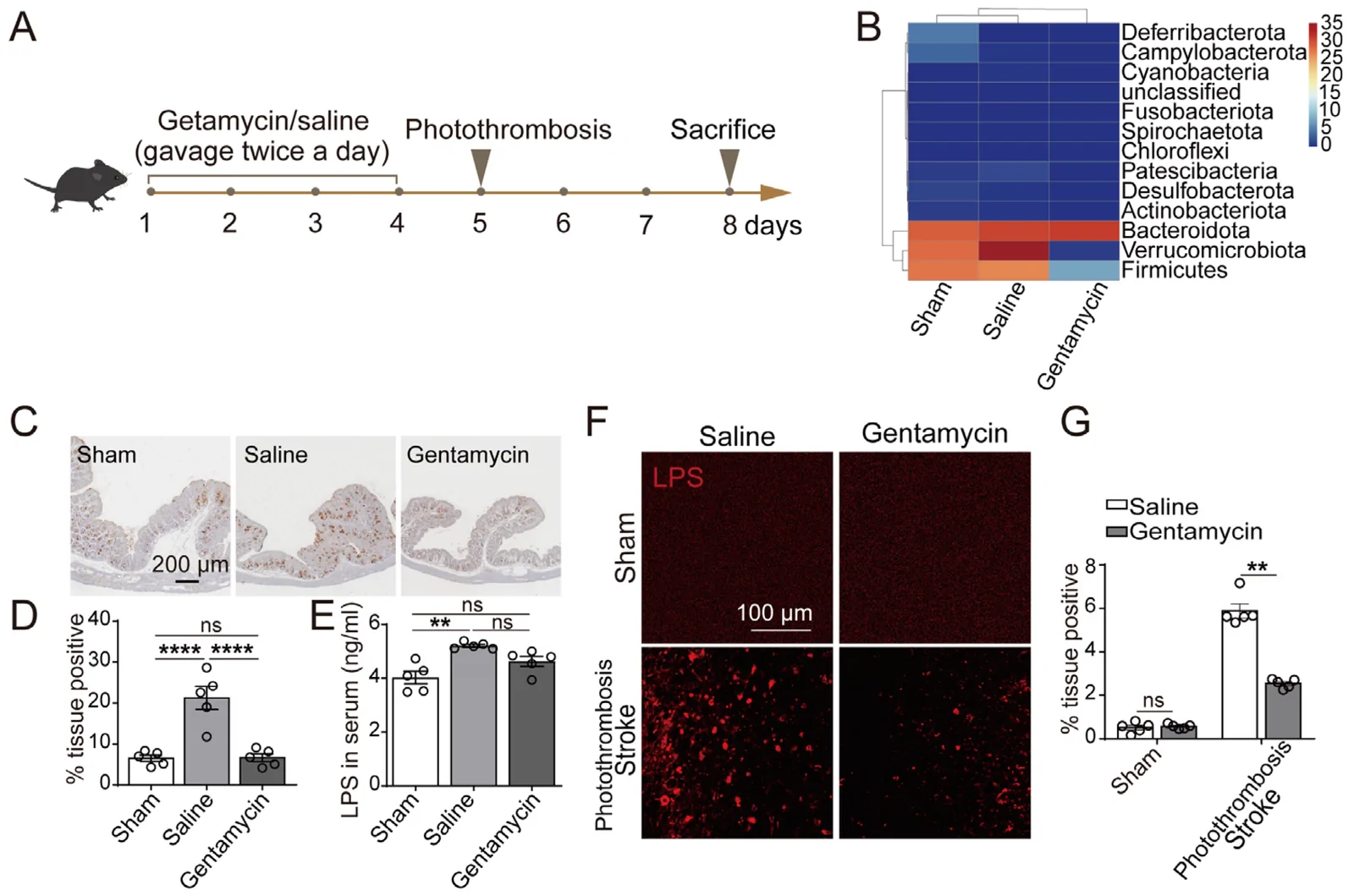
The detection of LPS levels in gut, blood and brain with gentamycin sulfate administration. (**A**) Timeline of gentamycin sulfate treatment. Mice were given gentamycin sulfate daily for 4 days before stroke was induced and sacrificed 3 days post-stroke. (**B**) Heatmap showing the relative abundance of gut microbiota at the phylum level in different groups. (**C**) IHC staining of LPS on colons in different groups. (**D**) The percentage of the positively stained area for LPS in the sham group, saline-treated and gentamycin-treated stroke groups (*n* = 5 per group). (**E**) The concentration of LPS in serum in the sham group, saline-treated and gentamycin-treated stroke groups (*n* = 5 per group). (**F**) Images of brain sections stained with LPS antibody after ischemic stroke in saline- and gentamycin-treated sham and stroke groups. (**G**) Quantitative analysis of LPS levels in the brain (*n* = 5 per group). Data are shown as mean ± S.E.M. ** *p* < 0.01, **** *p* < 0.0001.

To investigate whether gut microbial composition is associated with endogenous LPS levels following ischemic stroke, gentamycin sulfate was administered for 4 days prior to photothrombosis to deplete Gram-negative bacteria in the gut (Figure 2A). Heat map analysis of 16S rDNA amplicon sequencing revealed that gentamycin treatment significantly reduced the abundances of both *Verrucomicrobiota* (Gram-negative) and *Firmicutes* (Gram-positive) at 3 days after photothrombosis (Figure 2B). Consistently, gentamycin treatment significantly decreased LPS levels in the gut to levels close to those in the sham group (Figure 2C,D). Importantly, gentamycin treatment also significantly reduced LPS levels in cerebral tissues compared with the saline-treated stroke group (Figure 2F,G), while no significant difference was detected in serum LPS levels between the two groups (Figure 2E). Notably, this antibiotic-induced reduction in *Verrucomicrobiota* and LPS was accompanied by exacerbated stroke outcomes (Figure S2). Together, these data reveal that gentamycin treatment concurrently reduces *Verrucomicrobiota* abundance and LPS levels in the gut and brain, and that these changes are associated with worsened stroke outcomes, suggesting that LPS, together with other effects of antibiotic-induced gut microbiota alterations, is collectively associated with the worsened post-stroke prognosis.

### 3.3. Gentamycin-Induced Changes in LPS Levels Are Associated with Altered Microglial Polarization

**Figure 3:**
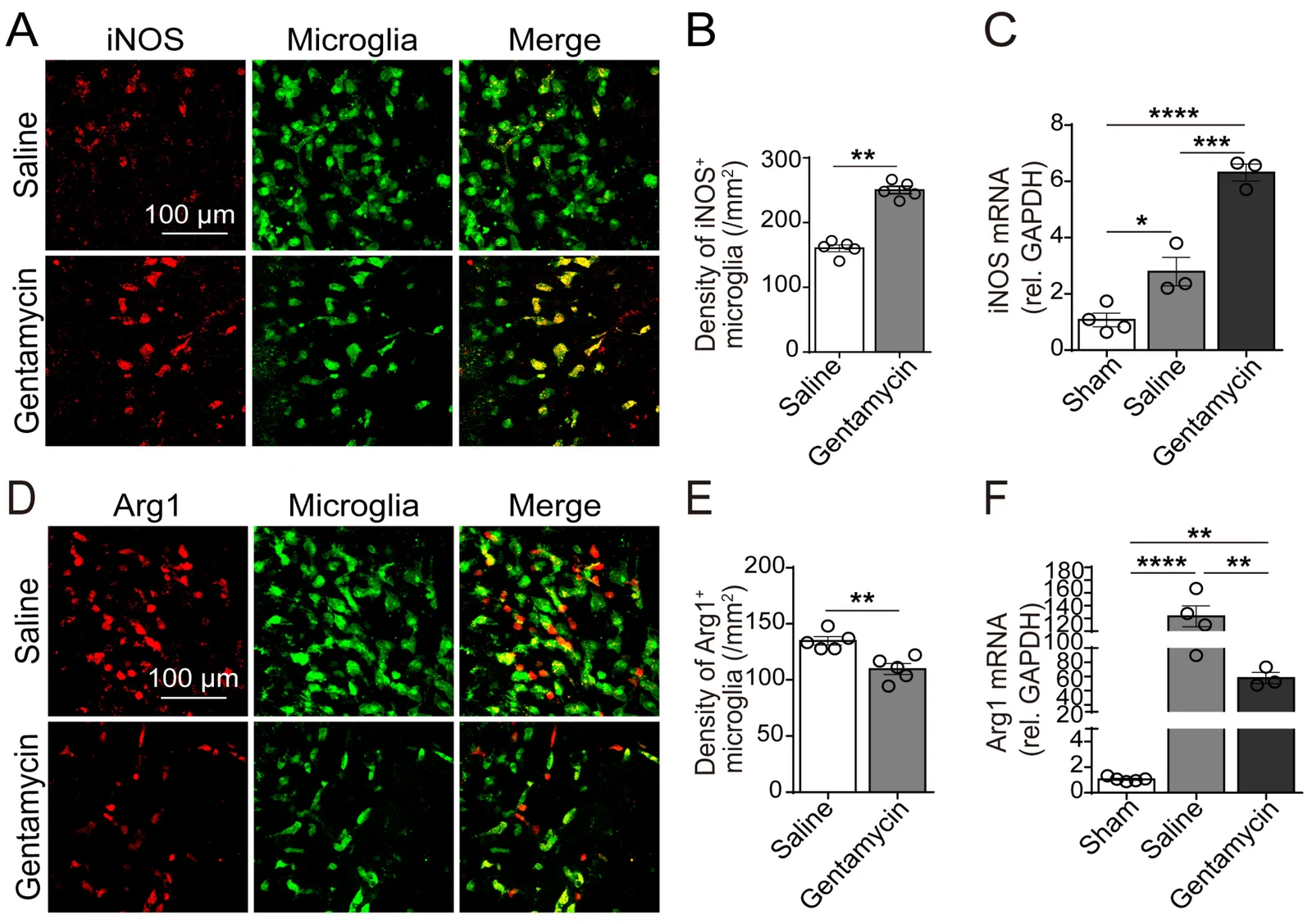
Assessment of iNOS and Arg1 after gentamicin administration in ischemic stroke. (**A**) Representative brain sections from the infarct border zone stained for iNOS across groups. (**B**) Quantification of iNOS-positive microglia in saline- and gentamicin-treated groups (*n* = 5 per group). (**C**) Relative iNOS mRNA levels in each group. (**D**) Representative images of brain sections from the infarct border zone immunostained for Arg1 in different groups after ischemic stroke. (**E**) The density of Arg1-positive microglia in different groups (*n* = 5 per group). (**F**) The gene expression of Arg1 in different groups. Data are shown as mean ± S.E.M. * *p* < 0.05, ** *p* < 0.01, *** *p* < 0.001, **** *p* < 0.0001.

Having observed that gentamycin treatment reduced *Verrucomicrobiota* and LPS levels, we reasoned that if LPS participates in post-stroke inflammation, then reducing LPS might be accompanied by changes in the brain’s inflammatory milieu. Since microglia are the primary immune cells in the brain and play a central role in neuroinflammation following ischemic injury, we next examined whether microglial polarization was affected by gentamycin treatment. IF staining of iNOS and Arg1 revealed that gentamycin treatment significantly increased the density of iNOS-positive microglia while decreasing the density of Arg1-positive microglia in the infarct border zone, consistent with their respective mRNA expression levels (Figure 3A–F). These observations indicate that gentamycin treatment, which reduced gut *Verrucomicrobiota* and LPS levels, was associated with a shift in microglial polarization toward a pro-inflammatory phenotype following ischemic stroke.

### 3.4. TLR4 Pathway Inhibition Is Associated with Altered Microglial Polarization Following Ischemic Stroke

**Figure 4:**
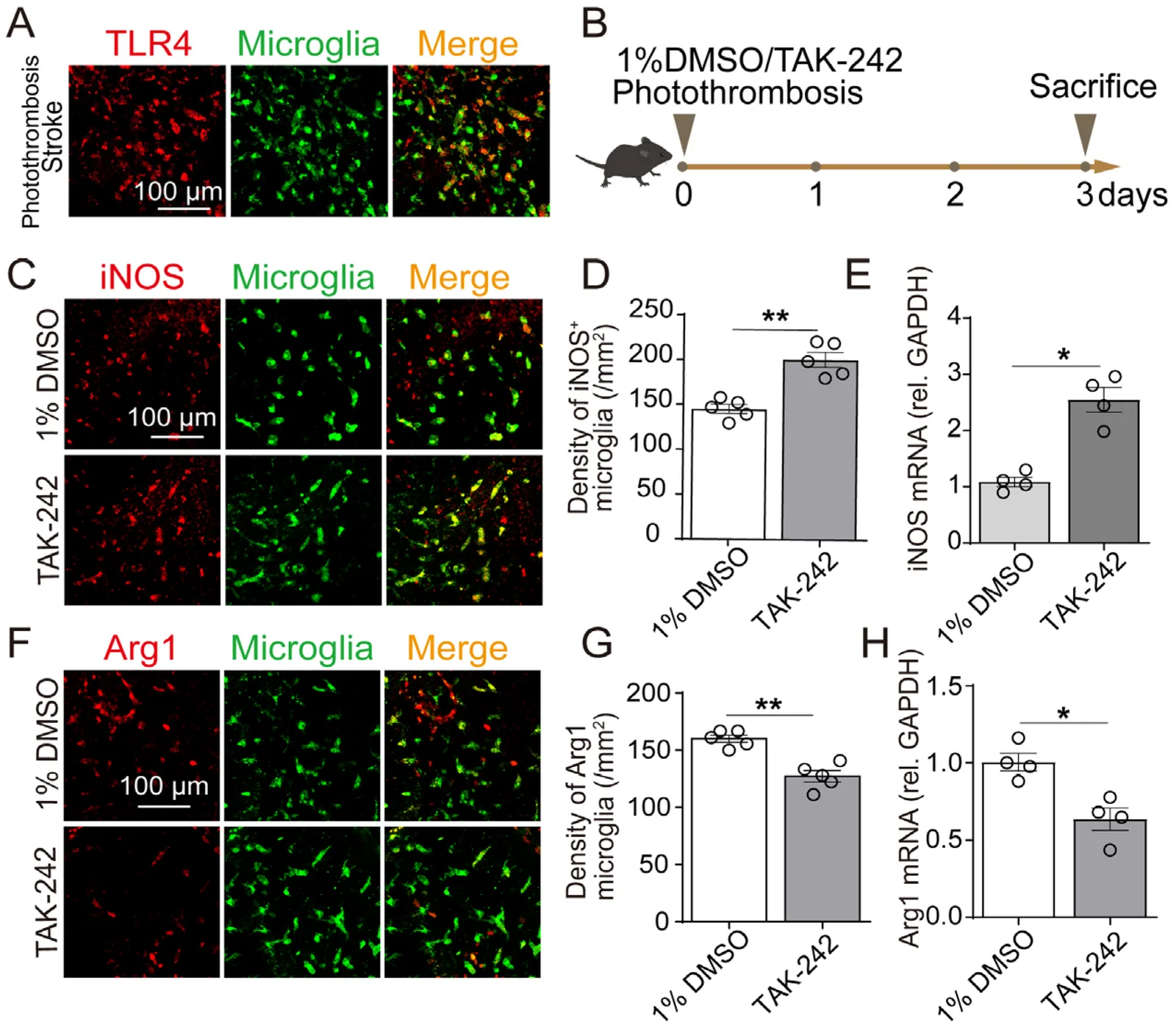
Detection of Arg1 and iNOS with TAK-242 administration after ischemic stroke. (**A**) The colocalization of TLR4 and microglia in the stroke group. (**B**) Diagram illustrating the TAK-242 and 1% DMSO treatment schedule; animals were sacrificed 3 days post-stroke. (**C**) Brain sections from the infarct border zone showing iNOS immunostaining across the TAK-242 and 1% DMSO groups. (**D**) iNOS-positive microglia counts in the TAK-242 and 1% DMSO groups (*n* = 5 per group). (**E**) iNOS mRNA expression levels in each group (*n* = 4 per group). (**F**) Representative images of brain sections from the infarct border zone immunostained for Arg1 in the TAK-242 and 1% DMSO groups following ischemic stroke. (**G**) The density of Arg1-positive microglia in TAK-242 and 1% DMSO groups after ischemic stroke (*n* = 5 per group). (**H**) The mRNA expression of Arg1 in 1% DMSO and TAK-242 groups (*n* = 4 per group). All comparisons in this figure were analyzed by the Mann–Whitney U test. Data are shown as mean ± S.E.M. * *p* < 0.05, ** *p* < 0.01.

As previously reported, LPS induces inflammatory responses via interaction with TLR4 [34,35]. We next examined whether the TLR4 signaling pathway is associated with microglial polarization in the ischemic brain. Our results showed that approximately 70–80% of TLR4 signals were colocalized with microglia in the stroke group (Figure 4A), suggesting that TLR4 is predominantly associated with microglia in the ischemic brain. To further investigate whether TLR4 signaling is associated with microglial responses, we examined the effects of TAK-242 (a TLR4 inhibitor) on microglial polarization (Figure 4B). Our results showed that TAK-242 treatment was associated with a significant increase in the density of iNOS-positive microglia (Figure 4C,D) and a decrease in the density of Arg1-positive microglia (Figure 4F,G). Consistently, qRT-PCR data revealed that TAK-242 administration was associated with increased iNOS mRNA expression and reduced Arg1 mRNA expression (Figure 4E,H). Thus, inhibition of the TLR4 pathway was associated with altered microglial polarization following ischemic stroke, a pattern consistent with the microglial phenotype changes observed after gentamycin treatment.

### 3.5. TLR4 Pathway Inhibition Is Associated with Inflammatory Responses and Impaired Functional Recovery

**Figure 5:**
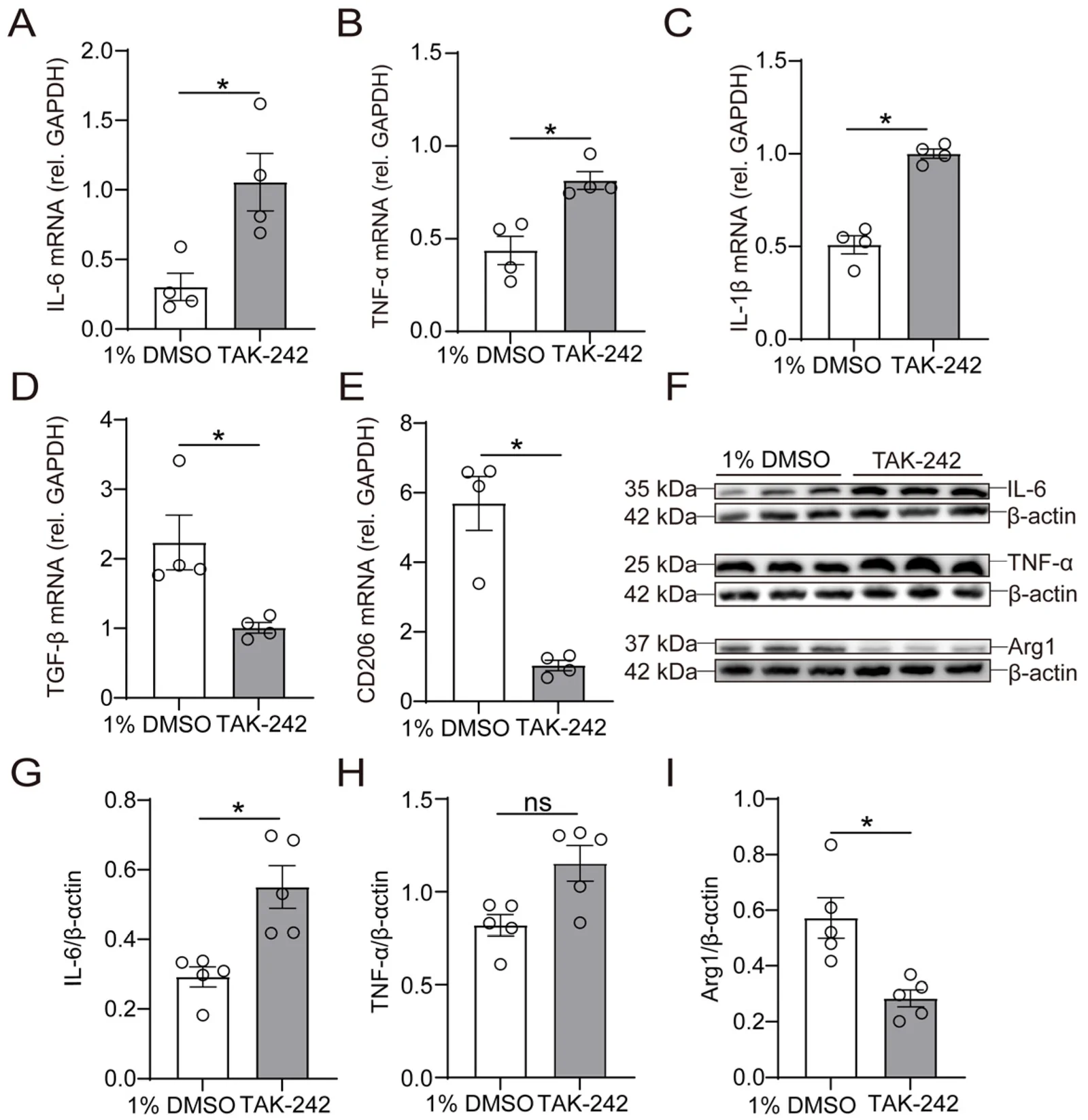
The gene and protein expressions of inflammatory factors with TAK-242 treatment. (**A**–**E**) mRNA expression levels of IL-6, TNF-α, IL-1β, TGF-β, and CD206 in each group (*n* = 4 per group). (**F**) Representative immunoblot bands for IL-6, TNF-α, Arg1, and the corresponding β-actin bands. Uncropped blots are shown in Figure S3. (**G**–**I**) Protein expression of IL-6, TNF-α, and Arg1 in each group (*n* = 5 per group). All comparisons in this figure were analyzed by the Mann–Whitney U test. Data are shown as mean ± S.E.M. * *p* < 0.05.

To further assess whether TLR4 pathway inhibition is associated with broader inflammatory responses and functional outcomes, we examined the effects of TAK-242 treatment on inflammatory cytokine expression, tissue damage, and behavioral performance following ischemic stroke. The results showed that TAK-242 treatment was associated with significantly increased mRNA levels of pro-inflammatory factors, including *IL-6*, *TNF-α*, and *IL-1β*, compared with the 1% DMSO group (Figure 5A–C). Conversely, TAK-242 treatment was associated with reduced mRNA levels of the anti-inflammatory factors *TGF-β* and *CD206* (Figure 5D,E). Consistent with these findings, TAK-242 administration was associated with increased IL-6 protein levels and decreased Arg1 protein levels, while TNF-α protein expression showed no significant change (Figure 5F–I and Figure S3A–C). Moreover, TAK-242 treatment was associated with increased neuronal degeneration and larger infarct volumes (Figure S4A–D), and behavioral tests further revealed that TAK-242-treated mice exhibited significantly decreased locomotor activity, forelimb grip strength, and rotarod performance compared with the 1% DMSO-treated stroke group (Figure S4E–H). Together, these findings demonstrate that TLR4 pathway inhibition is associated with a broad pro-inflammatory response, accompanied by aggravated tissue damage and behavioral deficits.

### 3.6. Exogenous LPS Produces Dose-Dependent Effects on Microglial Polarization

**Figure 6:**
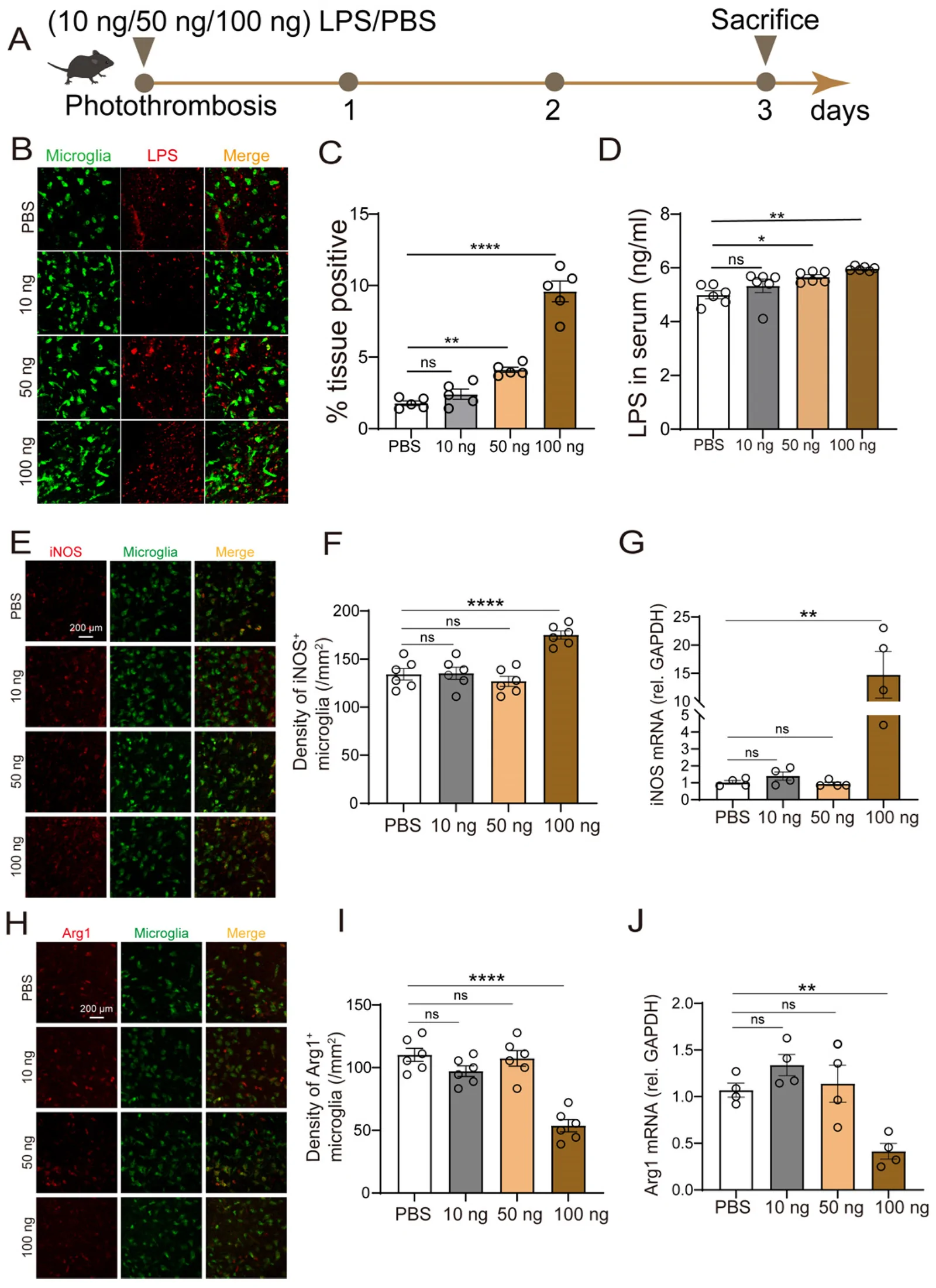
The IF staining and gene expression of Arg1 and iNOS with different doses of LPS treatment. (**A**) Experimental design for LPS treatment. The injection of LPS was performed before the modeling of ischemic stroke, and mice were sacrificed 3 days after stroke. (**B**) LPS staining in brain sections of CX3CR1GFP/+ mice following administration of different doses of LPS. (**C**) Quantitative analysis of LPS content in brain tissue (*n* = 5 per group). (**D**) Serum LPS concentrations in mice after treatment with different doses of LPS, measured by ELISA. (**E**) Brain sections from the infarct border zone showing iNOS immunostaining across groups. (**F**) iNOS-positive microglia counts in each group (*n* = 6 per group). (**G**) iNOS mRNA expression levels in each group (*n* = 4 per group). (**H**) Brain sections from the infarct border zone showing Arg1 immunostaining across groups. (**I**) Arg1-positive microglia counts in mice receiving different doses of LPS (*n* = 6 per group). (**J**) Arg1 mRNA expression levels in each group (*n* = 4 per group). Data are shown as mean ± S.E.M. * *p* < 0.05, ** *p* < 0.01, **** *p* < 0.0001.

LPS is widely used to induce pro-inflammatory responses in experimental models, yet whether endogenous LPS exerts similar effects in the post-ischemic brain remains unclear. To address this, we performed exogenous LPS supplementation experiments using doses based on the serum LPS levels detected after ischemic stroke (Figure 6A). We first measured LPS concentrations in the brain and serum at 3 days after stroke following different doses of LPS injection. The results showed that LPS levels in the brain were not significantly increased at 10 ng but were significantly elevated at 50 ng and 100 ng (Figure 6B,C); serum LPS concentrations showed a similar pattern (Figure 6D). We next examined the effects of these doses on microglial polarization. IF staining demonstrated comparable numbers of iNOS-positive microglia in the PBS, 10 ng, and 50 ng groups, whereas a marked increase was observed in the 100 ng group (Figure 6E,F). Conversely, Arg1-positive microglia were significantly decreased only in the 100 ng group, with no significant changes observed at 10 ng or 50 ng (Figure 6H,I). These findings were consistent with the mRNA expression levels of *iNOS* and *Arg1* (Figure 6G,J). Together, these data indicate that the effects of exogenous LPS on microglial polarization are dose-dependent, with detectable effects only at higher concentrations, whereas endogenous LPS levels remained relatively low after stroke.

### 3.7. LPS Exerts Dose-Dependent Effects on Neuroinflammatory Responses and Stroke Outcomes

**Figure 7:**
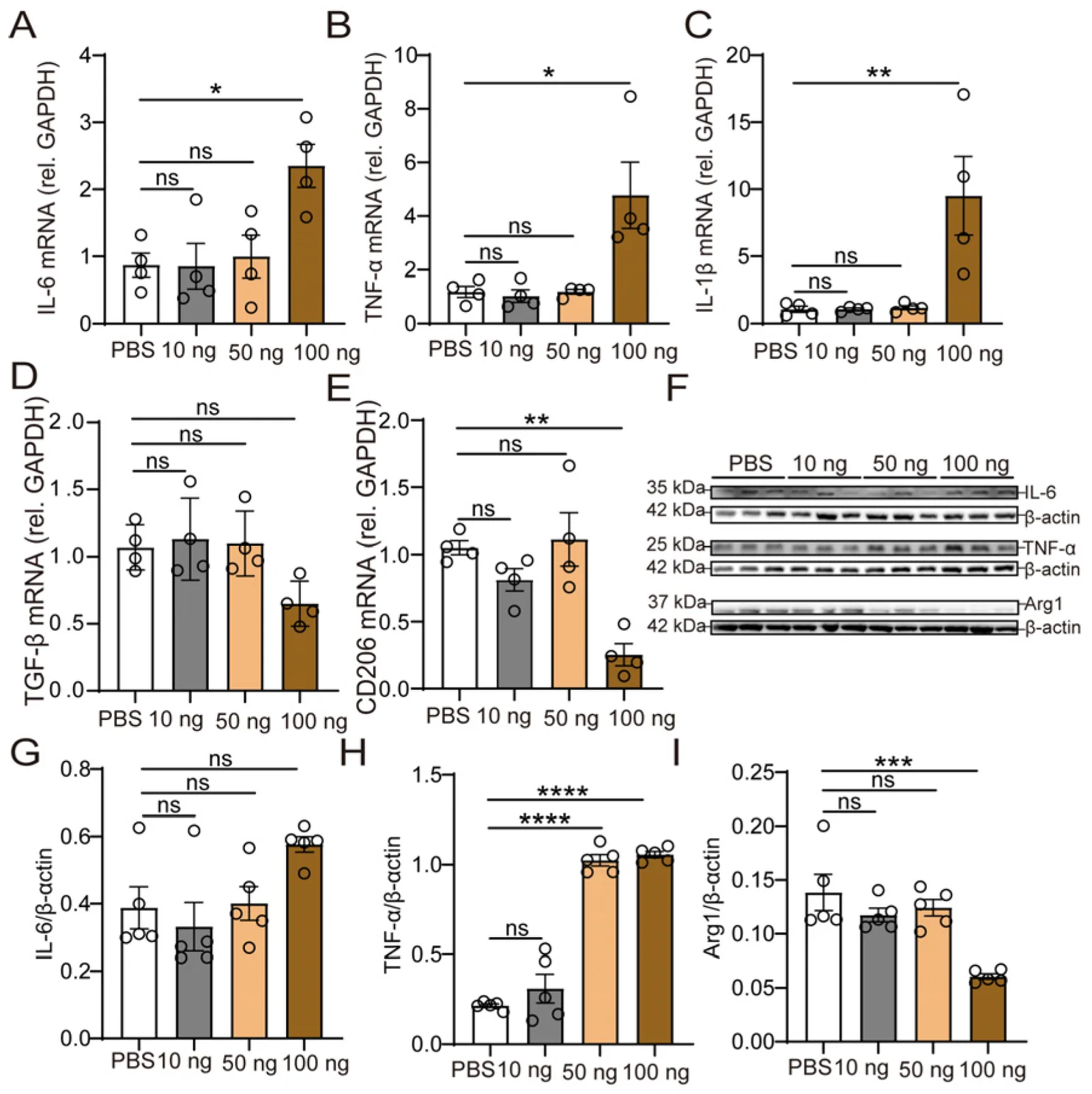
Expression of inflammatory mediators at mRNA and protein levels following LPS treatment after stroke. (**A**–**E**) mRNA expression of IL-6, TNF-α, IL-1β, TGF-β, and CD206 in each LPS dose group (*n* = 4 per group). (**F**) Immunoblot bands for IL-6, TNF-α, Arg1, and corresponding β-actin bands. Uncropped blots are shown in Figure S5. (**G**–**I**) Protein expression of IL-6, TNF-α, and Arg1 in each group (*n* = 5 per group). Data are shown as mean ± S.E.M. Data are shown as mean ± S.E.M. * *p* < 0.05, ** *p* < 0.01, *** *p* < 0.001, **** *p* < 0.0001.

To determine whether the dose-dependent effects of LPS extend beyond microglial polarization, we next examined inflammatory responses and functional outcomes following LPS administration. We measured the mRNA and protein levels of inflammatory cytokines following administration of varying doses of LPS after ischemic stroke. The qRT-PCR analysis showed that mRNA levels of *IL-6*, *TNF-α*, *IL-1β*, *TGF-β*, and *CD206* did not significantly differ among the PBS, 10 ng, and 50 ng groups (Figure 7A–E). Notably, the mRNA levels of *IL-6*, *TNF-α*, and *IL-1β* were significantly increased in the 100 ng group compared with the PBS group, whereas *CD206* mRNA was significantly decreased in the 100 ng group (Figure 7A–E). *TGF-β* mRNA showed no significant differences across all groups (Figure 7D). At the protein level, TNF-α was elevated in both the 50 ng and 100 ng groups compared with the PBS group, while IL-6 protein showed no significant differences among groups (Figure 7F–H and Figure S5A,B). Arg1 protein was significantly decreased only in the 100 ng group (Figure 7F,I and Figure S5C). Consistent with these inflammatory changes, high-dose LPS (100 ng) was associated with increased neuronal degeneration and infarct volumes (Figure S6A–D), as well as exacerbated behavioral deficits, whereas low doses (10 ng and 50 ng) had no detectable effects on these outcomes (Figure S6E–H). Together, these data indicate that exogenous LPS exerts dose-dependent effects on neuroinflammation and stroke outcomes, with significant effects observed only at higher concentrations, while endogenous LPS levels remained low.

## 4. Discussion

Previous studies showed that Type 2 diabetes is linked to alterations in gut microbiota composition and compromised integrity of the intestinal barrier, resulting in the migration of microbiota-derived LPS into the circulatory system [36]. Furthermore, LPS, arising from abundant Gram-negative bacilli of the gastrointestinal tract, is abundant in AD-affected brain neocortex and hippocampus and is considered to be an important initiator and/or significant contributor to inflammatory degeneration [37]. Consistently, our results showed elevated LPS levels in various tissues, including the gut, blood, and brain. In vivo, gentamycin treatment reduced the abundance of *Verrucomicrobiota* and also decreased LPS levels in the gut and brain following ischemic stroke. It is important to note that gentamycin treatment reduced LPS levels in the gut and brain without a detectable change in serum LPS. This apparent dissociation suggests that the relationship between circulating and tissue LPS is not a simple sequential cascade. Several factors may contribute to this observation, including the short half-life of circulating LPS, differences in detection sensitivity between ELISA and immunohistochemistry, and the potential involvement of local mechanisms independent of circulating LPS [38,39]. Further, our previous published work has directly demonstrated that *Akkermansia muciniphila* (currently the only identified species within the phylum *Verrucomicrobiota*) is beneficial for stroke outcomes. Notably, *Akkermansia muciniphila* was among the bacterial taxa significantly reduced following gentamycin treatment [40]. This suggests that the loss of *Akkermansia muciniphila* following gentamycin treatment may have a greater impact on stroke prognosis than the reduction in LPS alone. These findings are consistent with our overall interpretation that LPS is associated with, rather than linearly determining, post-stroke outcomes. Taken together, these results suggest that endogenous LPS might play critical roles in CNS diseases by being involved in neuroinflammatory responses.

LPS stimulates inflammatory responses by activating TLR4, which is expressed on multiple cell types in the brain, including microglia, neurons, and endothelial cells [36]. Among these, microglia are the brain-resident immune cells and play a central role in ischemic stroke pathophysiology [41], with the TLR4/NF-κB pathway being critically involved in their polarization [42,43]. In our study, approximately 70–80% of TLR4 signals colocalized with microglia, and administration of TAK-242, a TLR4 inhibitor, was associated with an increased density of pro-inflammatory microglia and a reduced density of anti-inflammatory microglia. This observation—that TLR4 blockade was associated with exacerbated inflammatory responses and worsened stroke outcomes—appears paradoxical, as TLR4 inhibition is conventionally expected to suppress inflammation. We propose that this may be explained by the dual downstream signaling of TLR4. TLR4 uniquely activates both the MyD88-dependent pathway (pro-inflammatory) and the TRIF-dependent pathway, which activates IRF3 and has been implicated in neuroprotection and tissue repair [44,45]. TAK-242 blocks both pathways simultaneously [46]; thus, while inhibiting MyD88 may reduce pro-inflammatory signaling, concurrent blockade of the protective TRIF-IRF3 axis may offset these benefits. This interpretation is supported by a recent study showing that TAK-242 exacerbated cognitive deficits in an Alzheimer’s disease model [47]. We acknowledge that TAK-242 may also exert indirect effects through other TLR4-expressing cells (e.g., neurons, astrocytes, endothelial cells), although our co-localization data indicate microglia as a primary target. Additionally, the intracerebroventricular route of administration may influence drug distribution and contribute to the observed effects. Previous studies have shown that TLR4 deficiency impairs microglial phagocytosis of degenerating axons [48], and TLR4 antagonism impairs recovery in intracerebral hemorrhage [49]. Accordingly, our data showed that TLR4 inhibition was associated with increased infarct volume and behavioral impairment at 3 days after stroke.

It is important to note that both the reduction in TLR4 signaling (via TAK-242) and its excessive activation (via high-dose exogenous LPS) were associated with worsened stroke outcomes, suggesting a potential non-linear or U-shaped relationship between LPS/TLR4 signaling and post-stroke outcomes. This pattern argues against a simple linear causal model and supports the interpretation that LPS-related signaling is correlated with, rather than linearly determining, post-stroke outcomes. Furthermore, both gentamycin (a broad-spectrum antibiotic) and TAK-242 (a TLR4 inhibitor) have non-specific effects that extend beyond LPS [11,50], and therefore the worsened outcomes observed cannot be attributed solely to LPS reduction. The combined effects of multiple microbial and immunological changes may contribute to the observed outcomes. Future studies using LPS-specific neutralization or cell type-specific TLR4 deletion are warranted to further dissect these relationships. Collectively, these findings suggest that endogenous LPS is associated with microglial polarization through the TLR4/NF-κB pathway, potentially influencing post-stroke outcomes.

There is evidence showing that the effects of LPS are concentration-dependent: high doses can reduce microvascular density, downregulate tight junction proteins, and increase endothelial permeability, whereas low doses exert opposite effects on these parameters [51,52]. At the cellular level, low-dose LPS promotes proliferation of pulmonary microvascular endothelial cells via Cdc42-mediated ERK signaling [53]. Consistently, LPS activates NF-κB in a dose-dependent manner: increasing concentrations drive p65 nuclear translocation in RAW 264.7 macrophages, with a maximal response at 100–300 ng/ml, while higher doses become cytotoxic [54]. Our LPS supplementation experiments are consistent with these findings, showing that high-dose LPS significantly increased pro-inflammatory cytokine expression, whereas anti-inflammatory cytokines exhibited an opposite trend. Notably, the serum LPS concentration in our stroke model (approximately 5.2 ± 0.05 ng/mL) was considerably lower than the levels typically used to induce acute pro-inflammatory responses (10–15 mg/kg in vivo) [55,56,57], but fell within the range associated with low-grade endotoxemia, which has been linked to endothelial senescence and chronic inflammation [58]. Thermoregulatory responses to LPS are also dose-dependent in mice, further supporting the concept that LPS exerts distinct biological effects depending on its concentration [59]. Moreover, beyond concentration, the structural variability of LPS across bacterial species may determine its biological activity: “good” versus “bad” LPS have been proposed [60], and LPS from *Akkermansia muciniphila* is structurally dissimilar to that of E. coli, inducing significantly lower IL-8 production in HT-29 cells [61]. It should be noted that the anti-*E. coli* LPS antibody (ab35654) used in our study was raised against *E. coli* J5 LPS, which recognizes the conserved inner core region of LPS, and may exhibit differential affinity across bacterial taxa [62,63]. The antibody has been reported to cross-react with *K. pneumoniae*, *S. sonnei*, and *S. typhimurium*, but its reactivity with LPS from *Verrucomicrobiota* has not been validated. Therefore, the immunostaining intensity should be interpreted as an indication of LPS immunoreactivity rather than a strict quantification of absolute LPS mass from specific species. Thus, the net biological effect of LPS is likely determined by both its concentration and its structural origin, warranting careful interpretation when evaluating its role in CNS disorders.

Notably, the transition from low-dose to high-dose effects appeared to occur between 50 ng and 100 ng in our study. While 10 ng and 50 ng failed to produce significant effects on most inflammatory parameters, 100 ng elicited robust responses. This pattern is consistent with the existence of a threshold for LPS activity, at which TLR4 signaling may shift from adaptive to maladaptive [64]. We acknowledge that intermediate doses between 50 ng and 100 ng were not examined, and therefore the precise threshold could not be defined. Importantly, the serum LPS concentration in our stroke model (approximately 5.2 ng/mL) was considerably lower than the doses that elicited robust inflammatory responses, suggesting that endogenous LPS may operate below the threshold required to trigger an exuberant inflammatory response. This is consistent with our overall conclusion that endogenous LPS is associated with a moderate inflammatory response rather than an acute, high-dose-like activation. Our findings suggest that even low concentrations of LPS, such as those observed in the post-stroke state, may exert significant biological effects, potentially through chronic low-grade inflammation rather than acute toxicity. This concentration-dependent duality should be carefully considered in future studies on the role of LPS in CNS pathophysiology.

It should be noted that the concurrent elevation of LPS in the blood and the presence of LPS immunoreactivity in the ischemic brain may reflect a secondary phenomenon associated with systemic inflammation. Bacterial LPS induces the release of inflammatory mediators that enter the bloodstream and can interact with CNS structures, modifying blood–brain barrier function and inducing aseptic neuroinflammation [65]. For example, low-grade endotoxemia is characterized by chronic, non-septic elevation of circulating LPS, and gut dysbiosis is a major determinant of this condition [66]. Our data demonstrate a spatial correlation between these events but cannot establish a causal relationship. Nevertheless, this correlation suggests that LPS may represent one of the biological mechanisms linking gut microbiota changes to CNS pathology following ischemic stroke, alongside other potential mediators such as microbial metabolites and immune cell trafficking. It is also important to acknowledge that gut microbiota characterization is inherently complex, influenced by diet, housing environment, and substantial inter-individual variability [67], and that investigating CNS pathologies from this perspective therefore presents significant practical challenges. Still, our findings suggest that LPS may serve as an accessible peripheral biomarker for monitoring post-stroke neuroinflammation. This study provides a perspective for future investigations into the gut–brain axis in ischemic stroke, rather than a definitive conclusion.

## 5. Conclusions

Our findings reveal a spatial correlation between gut microbial changes and cerebral LPS immunoreactivity, suggesting that endogenous LPS may serve as a correlative marker linking gut microbiota to neuroinflammation after ischemic stroke. We acknowledge the limitations regarding antibody specificity and source tracing, and future studies are warranted to further elucidate these aspects. Nevertheless, this study provides an observational foundation for understanding the relationship between gut microbiota and post-stroke neuroinflammatory responses.

## Data Availability

The sequencing data presented in this study are openly available in the NCBI Sequence Read Archive (SRA) under accession numbers PRJNA990935 and PRJNA1073280. The raw data supporting the other findings of this study are available from the corresponding authors upon reasonable request.

## Supplementary Materials

The following supporting information can be downloaded at: https://www.mdpi.com/article/10.3390/cells15191819/s1, Supplementary Methods S1. Measurement of cerebral infarct volumes; Supplementary Methods S2. Behavioral tests; Supplementary Methods S3. Fluoro-Jade C (FJC) staining [68]. Figure S1: Ischemic stroke induced an increase in LPS levels in the brain; Figure S2: The effects of gentamycin on stroke outcomes. Figure S3: The full uncropped blot images in PVDF membranes within TAK-242 treatment. Figure S4: TAK-242 treatment led to worse stroke outcomes. Figure S5: The full uncropped blot images in PVDF membranes within different doses of LPS treatment. Figure S6: Different doses of LPS have different effects on stroke outcomes. Table S1: Sequences of primers used in this study.

## Abbreviations

The following abbreviations are used in this manuscript:

MGBA	Microbiota–gut–brain axis
LPS	Lipopolysaccharide
CNS	Central nervous system
BBB	Blood–brain barrier
FMT	Fecal microbiota transfer
TMAO	Trimethylamine-N-oxide
SCFAs	Short-chain fatty acids
AD	Alzheimer’s disease
GFP	Green fluorescent protein
IHC	Immunohistochemistry
IF	Immunofluorescence
