# Supplementary material for: Correlation Between Gut Microbiota Shifts, Lipopolysaccharide Levels, and Neuroinflammatory Responses Following Ischemic Stroke
Source: Cells. 2026 Oct 6;15(19):1819. doi: 10.3390/cells15191819 (PMC13664211; doi:10.3390/cells15191819)
Supplement: Supplementary file 1 [file cells-15-01819-s001.zip › cells-4579293-supplementary.pdf]

Supplementary Materials

Correlation Between Gut Microbiota Shifts, Lipopolysaccharide Levels, and Neuroinflammatory Responses Following Ischemic Stroke

Kemin Li [1,*], Wancong Ding [2], Shuang Wang [2], Gaowei Pan [2] and Shengxiang Zhang [2,*]

[1] School of Food Science and Engineering, Xinjiang Institute of Technology, No. 1 Xuefu Road West, Aksu 843100, China
[2] Gansu Key Laboratory of Biomonitoring and Bioremediation for Environmental Pollution, School of Life Sciences, Lanzhou University, No. 222 South Tianshui Road, Lanzhou 730000, China; dingwc2023@lzu.edu.cn (W.D.); wshuang2025@lzu.edu.cn (S.W.); pangw20@lzu.edu.cn (G.P.)
* Correspondence: 2025032@xjit.edu.cn (K.L.); sxzhang@lzu.edu.cn (S.Z.)

Supplementary methods

1.Measurement of cerebral infarct volumes

Nissl's staining was employed to evaluate the infarct volumes. Brain sections (30 μm) were mounted onto gel-coated slides and subsequently stained with Cresyl Violet for 10 min at 65°C. The sections were then rinsed with distilled water, followed by dehydration using a gradient of alcohol concentrations (70%, 85%, 95%, and 100%). Afterward, the sections were cleared with xylene for 3 min, and slides were mounted using neutral balsam. Photography was performed using an Olympus DP72 microscope. Infarct volumes were estimated using a systematic sampling approach appropriate for the photothrombotic model. Coronal sections were collected at 30 μm thickness, and every third section (i.e., one section from each set of three consecutive sections, with a sampling interval of 90 μm) was selected for infarct area measurement. The infarct area of each selected section was measured using ImageJ software. The infarct volume was calculated as: infarct volume = Σ (a1 + a2 + … + an) × 30 × 3, where a1–an represent the infarct areas of the selected sections, 30 μm is the section thickness, and the factor of 3 accounts for the sampling interval (every third section). All volumes are expressed in mm^3. This method was applied consistently across all experimental groups to allow relative comparison of infarct size.

2.Behavioral tests

To assess motor function deficits, endurance and forelimb grip strength, three tests were conducted: the open field test, rotarod test, and forepaw grip strength test, respectively. In the open field test, mice were placed in a test chamber (25 cm (length) x 25 cm (width) x 30 cm (height), Jiliang) for 35 min, and data were collected during the last 30 min of the session. Endurance was assessed using a rotarod apparatus (TECHMAN; ZB-200) with the rotarod speed increasing from 10 rpm to 40 rpm over 5 min. Mice were trained three times daily for three days before surgery, and those capable of remaining on the rod for >200 seconds were selected for subsequent experiments. The duration each mouse remained on the rotarod was recorded. Forelimb grip strength was measured using a force transducer (BL-420S; TECHMAN). Mice were placed with their forelimbs on a specialized tripod, and gentle traction was applied to their tails until their forelimbs lost grip. The maximum grip strength at this point was then recorded.

3.Fluoro-Jade C (FJC) staining

Fluoro-Jade C (FJC) staining was employed to identify degenerating neurons [1]. Brain sections (30 μm) were obtained using a vibrating microtome and then mounted onto gelled slides. The slides were immersed in 0.06% potassium permanganate solution in darkness with gentle shaking for 10 min at room temperature. After rinsing with distilled water, the slides were transferred to a solution of 0.0001% FJC (dissolved in 0.1% acetic acid) for 20 min at room temperature in darkness. Following staining, the slides were rinsed with distilled water and dried at 37°C for 30 min. Then, the slides were briefly immersed in 100% ethanol for 2 min, followed by three changes of xylene. Finally, the slides were mounted using DPX (Sigma). Images were conducted using an Olympus DP72 microscope with 488 nm exciting light.

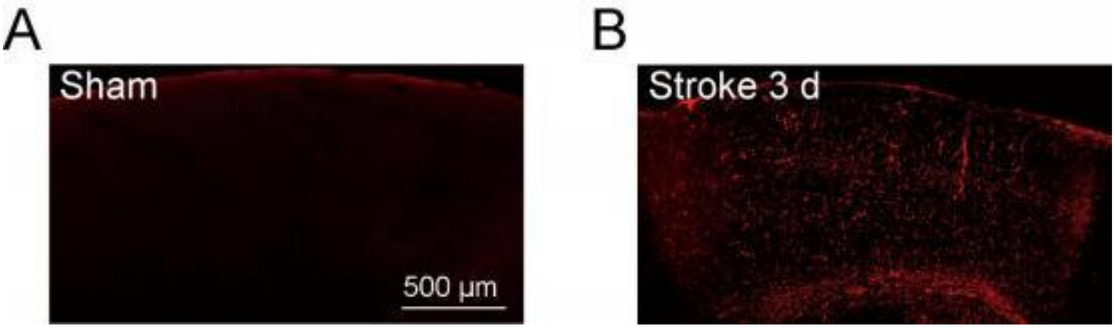

Figure S1. Ischemic stroke induced an increase of LPS levels in brain. (A) The IF staining of LPS in sham group. (B) The IF staining of LPS in stroke group.

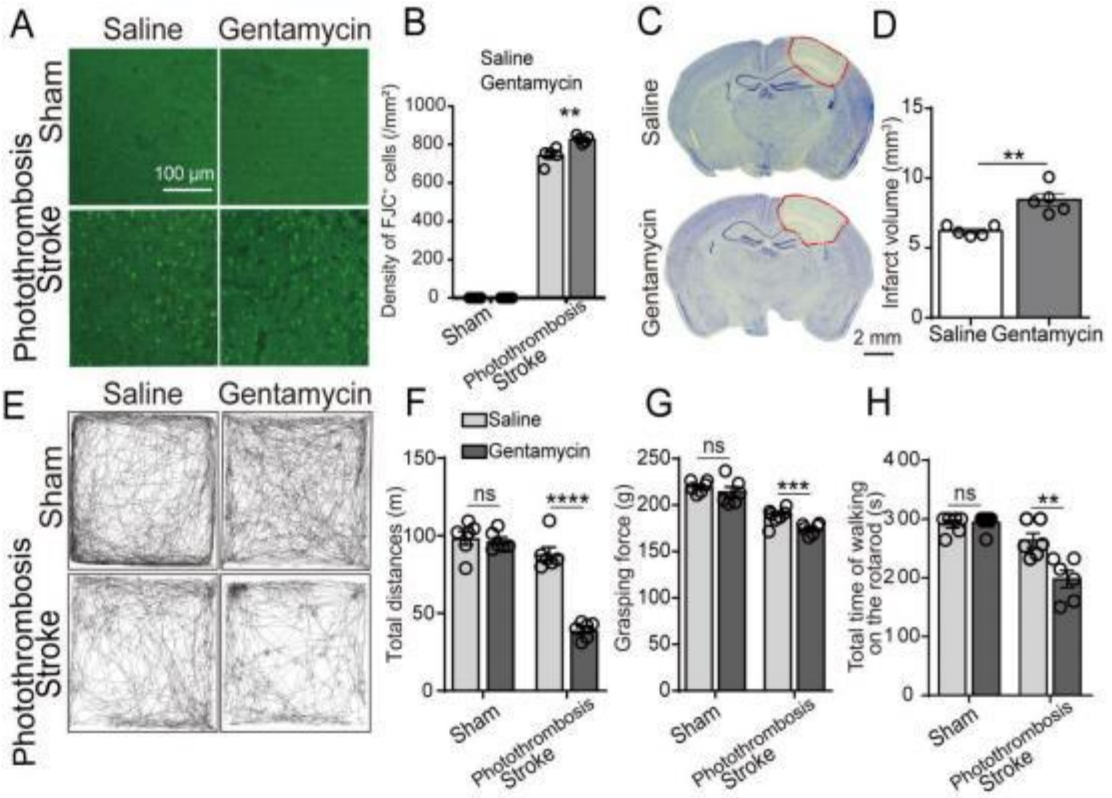

Figure S2. The effects of gentamycin on stroke outcomes. (A) Representative images of FJC-stained brain sections in saline and gentamycin treated sham and stroke group. (B) The histogram showing the density of FJC in the boundary zone of infarction (n=5 per group). (C) Representative images of Cresyl Violet-stained brain sections, and the infarct areas are showed in red dashed boxes. (D) The infarct volumes of mice in saline and gentamycin groups after ischemic stroke (n=5 per group). (E) Trajectory diagrams of open field tests in saline and gentamycin groups. (F) The histogram showing the total distances of mice moved in open field in different groups (n=6 per group). (G) The grasping forces of forelimbs in sham and stroke groups (n=6 per group). (H) The histogram showing the total time of walking on the rotarod in different groups (n=6 per group). Data are shown as mean ± S.E.M. **p < 0.01, ***p < 0.001, ****p < 0.0001.

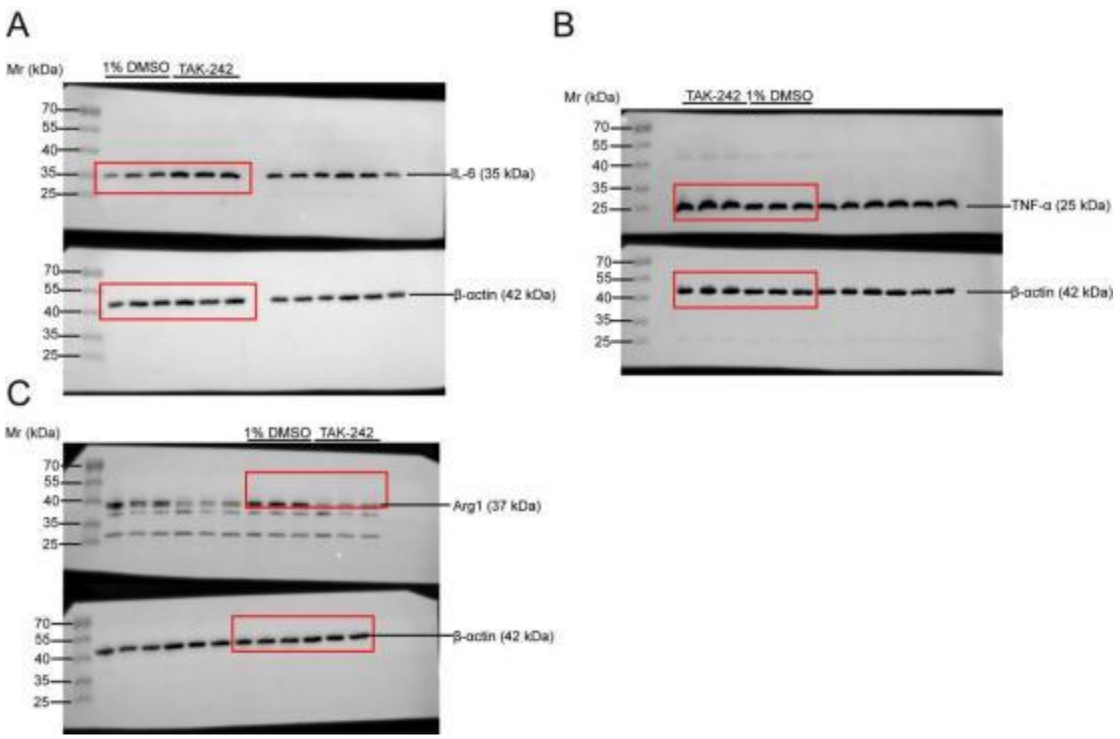

Figure S3. The full uncropped blots images in PVDF membranes within TAK-242 treatment. (A-C) The images show the uncropped images of IL-6, TNF-α, Arg1 and their corresponding bands of β-actin in different groups in PVDF membranes. Molecular-weight markers are indicated on the left side of each blot. The images of the target protein and its corresponding β-actin are from the same gel. The images in red box are the sources of the protein bands in the manuscript (Figure 5F). No lanes were removed or rearranged in the original blots.

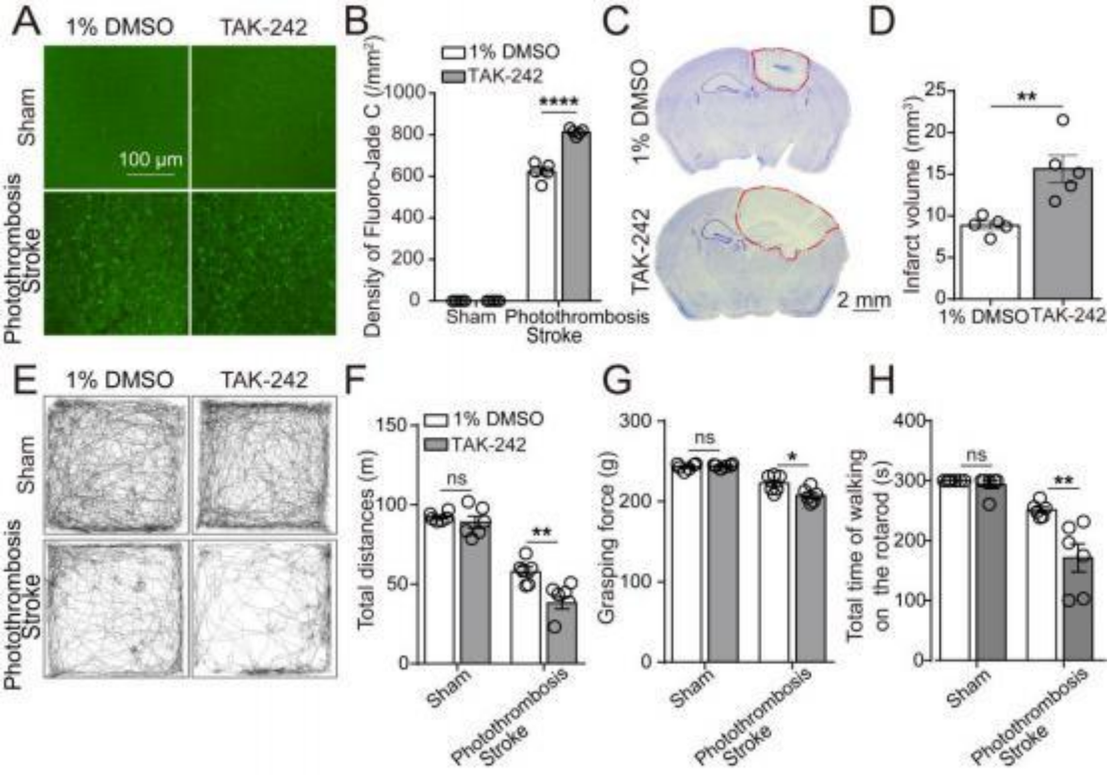

Figure S4. TAK-242 treatment led to worse stroke outcomes. (A) Images showing FJC-positive cells in TAK-242 and 1% DMSO groups after ischemic stroke. (B) The density of FJC-positive cells in TAK-242 and 1% DMSO groups after ischemic stroke (n=5 per group). (C) The infarct volumes were measured by Nissl's staining, and infarct areas were showed in red dotted boxes. (D) The infarct volumes in 1% DMSO and TAK-242 groups (n=5 per group). (E) Trajectory diagrams of the open field tests in 1% DMSO and TAK-242 groups. (F) The total distances of mice moved during open field in different groups (n=6 per group). (G) The grasping forces of forelimbs in different groups (n=6 per group). (H) The time of mice walking on the rotarod in different groups (n=6 per group). Data are shown as mean ± S.E.M. $*p < 0.05$, $**p < 0.01$, $****p < 0.0001$.

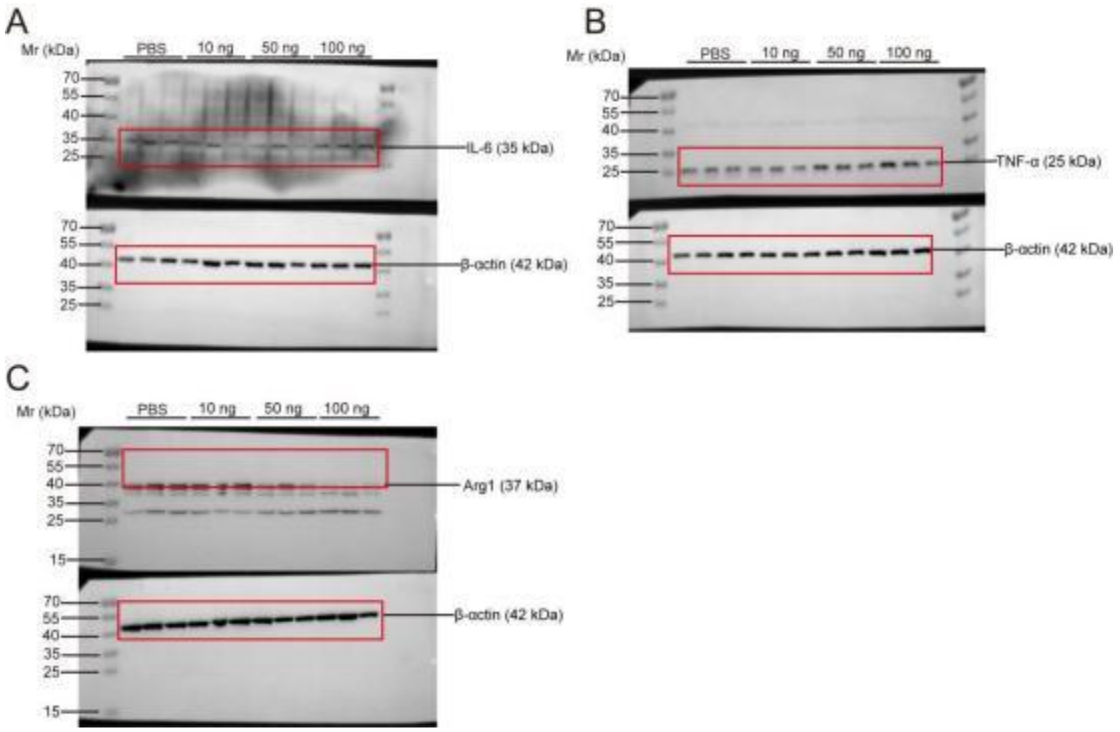

Figure S5. The full uncropped blots images in PVDF membranes within different doses of LPS treatment. (A-C) The images show the uncropped images of IL-6, TNF-α, Arg1 and their corresponding bands of β-actin in different groups in PVDF membranes. Molecular-weight markers are indicated on the left side of each blot. The images of the target protein and its corresponding β-actin are from the same gel. The images in red box are the sources of the protein bands in the manuscript (Figure. 7F). No lanes were removed or rearranged in the original blots.

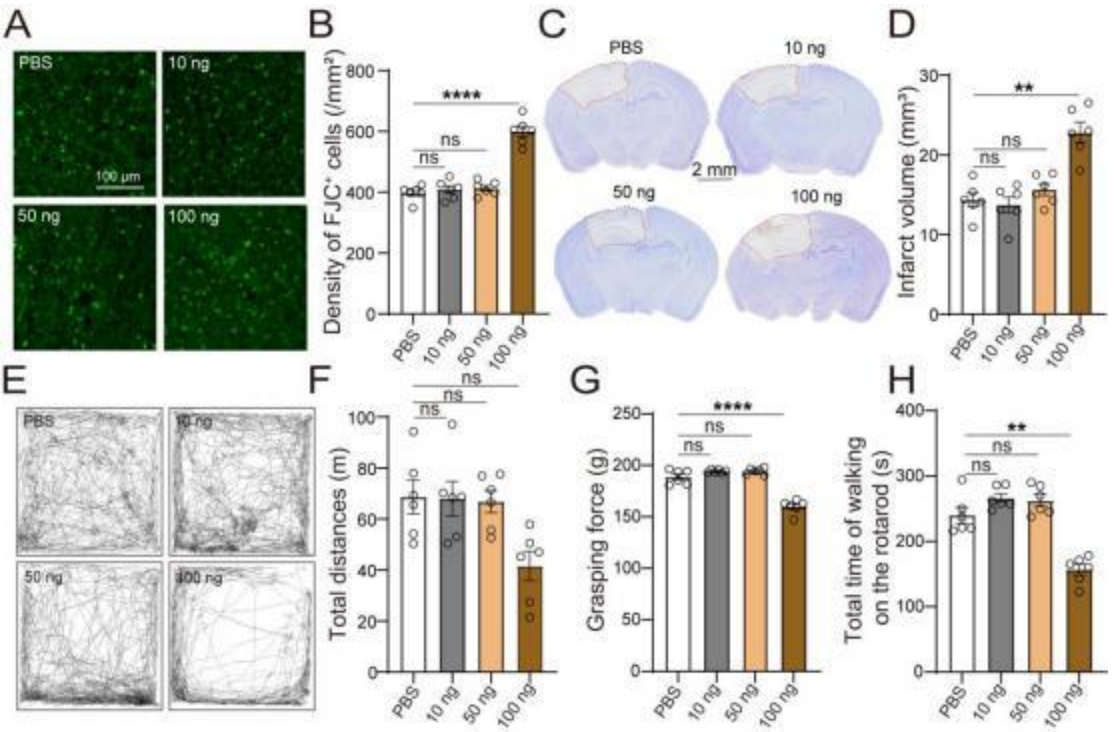

Figure S6. Different doses of LPS have different effects on stroke outcomes. (A) Images showing FJC-stained sections in different groups after ischemic stroke. (B) The density of FJC-positive cells in different groups (n=6 per group). (C) The Nissl's staining was used to measure the infarct volumes, and infarct areas were showed in red dotted boxes. (D) The infarct volumes of mice in different groups of LPS supplementation after stroke (n=6 per group). (E) Trajectory diagrams of the open field tests in different groups. (F) The histogram showing the total distances of mice moved in open field in different groups (n=6 per group). (G) Grasping forces of forelimbs in different groups (n=6 per group). (H) The total time of walking on the rotarod in different groups (n=6 per group). Data are shown as mean ± S.E.M. **$p < 0.01$, ****$p < 0.0001$.

Supplementary Tables

Table S1. Sequences of primers used in this study.

	Gene name	Sequences ofthe primers (5' – 3')
Inflammatory factors	*TGF-β*	*F: GGCGATACCTCAGCAACCG*<br>*R: CTAAGGCGAAAGCCCTCAAT*
	TNF-α	*F: GACGTGGAACTGGCAGAAGA*<br>*R: ACTGATGAGAGGGAGGCCAT*
	IL-1β	*F: CCTCGTGCTGTCGGACCCATA*<br>*R:CAGGCTTGTGCTCTGCTTGTA*
	IL-6	*F: TAGTCCTTCCTACCCCAATTTC*<br>*R: TTGGTCCTTAGCCACTCCTTC*
	Arg1	*F: TCACCTGAGCTTTGATGTCG*<br>*R: CTGAAAGGAGCCCTGTCTTG*
	CD206	*F: GAGGGAAGCGAGAGATTATGGA*<br>*R: GCCTGATGCCAGGTTAAAGCA*
	iNOS	*F: GCGAAAGGTCATGGCTTCAC*<br>*R: CTGGTCCATGCAGACAACCT*
	GAPDH	*F:CGTGCCGCCTGGAGAAACCTG*<br>*R:AGAGTGGGAGTTGCTGTTGAAGTCG*

Supplementary References

[1] L. C. Schmued, C. C. Stowers, A. C. Scallet, and L. Xu, "Fluoro-Jade C results in ultra high resolution and contrast labeling of degenerating neurons.," *Brain Res.*, vol. 1035, no. 1, pp. 24–31, Feb. 2005, doi: 10.1016/j.brainres.2004.11.054.

References cited in the Supplementary Materials are included in the main reference list; see ref. [68] in the main text.
